# Preparation of Curcumin Hydrogel Beads for the Development of Functional *Kulfi*: A Tailoring Delivery System

**DOI:** 10.3390/foods11020182

**Published:** 2022-01-11

**Authors:** Minaxi Sharma, Baskaran Stephen Inbaraj, Praveen Kumar Dikkala, Kandi Sridhar, Arjun Naik Mude, Kairam Narsaiah

**Affiliations:** 1 Central Institute of Post-Harvest Engineering and Technology, Ludhiana 141 004, India; minaxi86sharma@gmail.com (M.S.); dikkalapraveenkumar@gmail.com (P.K.D.); arjunnaik133@gmail.com (A.N.M.); 2 Department of Food Science, Fu Jen Catholic University, New Taipei City 242 05, Taiwan; sinbaraj@yahoo.com or

**Keywords:** curcumin, hydrogel beads, encapsulation efficiency, in vitro release, fortification, functional *Kulfi*, sensory evaluation

## Abstract

Curcumin has been demonstrated to have biological activities and its fortification in food products is an important strategy to deliver bioactive ingredients at target sites. However, studies have documented a curcumin low bioavailability and low intake. Hence, combining functional ingredients with food should be needed to prevent widespread nutrient intake shortfalls and associated deficiencies. Thus, curcumin was encapsulated in calcium-alginate and their characteristics as well as in vitro release behavior of curcumin hydrogel beads (CHBs) was studied. Moreover, CHBs were fortified in development of functional *Kulfi* and their quality characteristics were studied. The encapsulation efficiency was up to 95.04%, indicating that most of the curcumin was entrapped. FTIR shifts in the bands were due to the replacement of sodium ions to the calcium ions. In vitro release (%) for CHBs was found to be 67.15% after 2 h, which increased slightly up to 67.88% after 4 h. The average swelling index of CHBs was found to be 10.21 to 37.92 from 2 to 12 h in PBS (pH 7.40). Control and *Kulfi* fortified with CHBs showed no significant difference (*p* > 0.05) in colour (L = 73.03 and 75.88) and the melting rate (0.88 mL/min and 0.63 mL/min), respectively. Standard plate count was reduced in the *Kulfi* fortified with CHBs (13.77 × 10^4^ CFU/mL) with high sensory score for overall acceptability (8.56) compared to the control (154.70 × 10^4^ CFU/mL). These findings suggested the feasibility of developing CHBs to mask the bitterness, enhance the solubility, and increase the bioavailability in gastrointestinal conditions. Additionally, *Kulfi* could be a suitable dairy delivery system for curcumin bioactive compounds.

## 1. Introduction

Natural antioxidants from plants are important for cancer prevention due to their capacity to neutralize free radicals in the human body. This is the reason why many traditional nutraceutical foods have been developed by combining food with functional ingredients. Consumer demand for health-promoting foods containing natural antioxidants are growing with a particular interest in natural functional ingredient(s) [1]. Turmeric (*Curcuma longa*) is a culinary spice and has a very long medicinal history that started nearly 4000 years ago. The major advantage of curcumin is that it lowers the toxicity even when it is taken in relatively high doses. However, a study addressed the low curcumin bioavailability in serum or tissue after oral administration [2].

The low bioavailability of curcumin resulted in poor solubility (under acidic and/or neutral conditions) due to poor absorption, chemical instability, rapid metabolism, and rapid systemic elimination [3,4]. Therefore, curcumin bioavailability studies, particularly delivery systems, are needed to improve the solubility of curcumin and facilitate the targeted release of curcumin within the gastrointestinal environment. The novel way to improve the curcumin bioavailability is by the application of encapsulation technology [5]. Generally, spray drying is the most commonly used method for the encapsulation of active ingredients; however, spray drying is an expensive method and needs a lot of time. This indicated a need to develop an alternate encapsulation method; thus, we developed an in-house microencapsulator [6,7,8].

Dairy desserts are well accepted by a wide variety of age groups and can be formulated with several functional ingredients. For instance, *Kulfi* is a popular traditional frozen Indian dairy dessert, which resembles the same composition as ice cream (also called a traditional Indian ice cream) with concentrated sweetened milk [9,10]. To date, no report has been highlighted to increase the bioavailability of dairy products (particularly, *Kulfi*) using encapsulated curcumin hydrogel beads. Moreover, our experience in working with food product development and microencapsulation was collectively motivated for this research.

Therefore, the objective of this study was to develop calcium-alginate hydrogel beads for the encapsulation of curcumin by ionotropic gelation method. Moreover, characteristics and in vitro release behavior of curcumin hydrogel beads (CHBs) were studied. To increase the functionality of CHBs, we developed CHBs-fortified functional *Kulfi* and studied its quality characteristics, including physical, microbial, and sensory attributes. The present study outcomes are expected to raise the possibility of using a microencapsulation system in fortification of curcumin bioactive compounds and application in dairy foods without affecting their quality and sensory acceptability.

## 2. Materials and Methods

### 2.1. Materials

Curcumin (food-grade; 98%) and sodium alginate (food-grade; 95%; molecular weight: 216.12 g/mol) were obtained from SD Fine Chemicals (Mumbai, Maharashtra, India). Liquid soy lecithin and whey protein concentrate (80%) were purchased from Sonic Biochem Extractions Ltd. (Indore, Madhya Pradesh, India) and Davisco Foods International (Le Sueur, MN, USA), respectively. Calcium chloride (97%) and the rest of chemicals or reagents used were of analytical/food grade and were procured from Sigma-Aldrich^®^ (Mumbai, Maharashtra, India). Other ingredients for *Kulfi* preparation were procured from Verka Milk plant, Ludhiana, Punjab, India.

### 2.2. Preparation of Curcumin Emulsions

To formulate coarse emulsion, curcumin (4%) and lecithin (0.50%), an emulsifier, were dissolved in distilled water by slow dissolution at room temperature under mechanical stirring using high-speed mechanical stirrer with a 3-bladed propeller at 1600 rpm (IKA, Staufen, Germany) for 10 min, followed by addition of stabilizer, whey protein concentrate (2%), by stirring (1600 rpm/10 min) and then sonicated (Vibra cell, VCA 500, Sonics & Materials Inc., Newtown, CT, USA) for 60 min (10 sec on/off). Finally, the obtained coarse emulsion was sonicated for 20 min to form fine emulsion and stored in amber colored scintillation vials at 4 °C for further experimental analysis.

### 2.3. Characterization of Emulsions

#### Particle Size Distribution and Apparent Viscosity

The average particle size distribution of the sample was measured by a nanoparticle size analyser (Malvern Panalytical Ltd., Malvern, UK) according to Kairam, et al. [8], while the apparent viscosity of samples was recorded by a dynamic shear rate rheometer (Physica HBR 101, Anton Paar, Graz, Austria) at 25 °C with a sample volume of 5 mL operating at varying shear rates from 0.01 to 300 (1/s) by using a 50 mm stainless steel parallel plate.

### 2.4. Encapsulation Process

**Figure 1:**
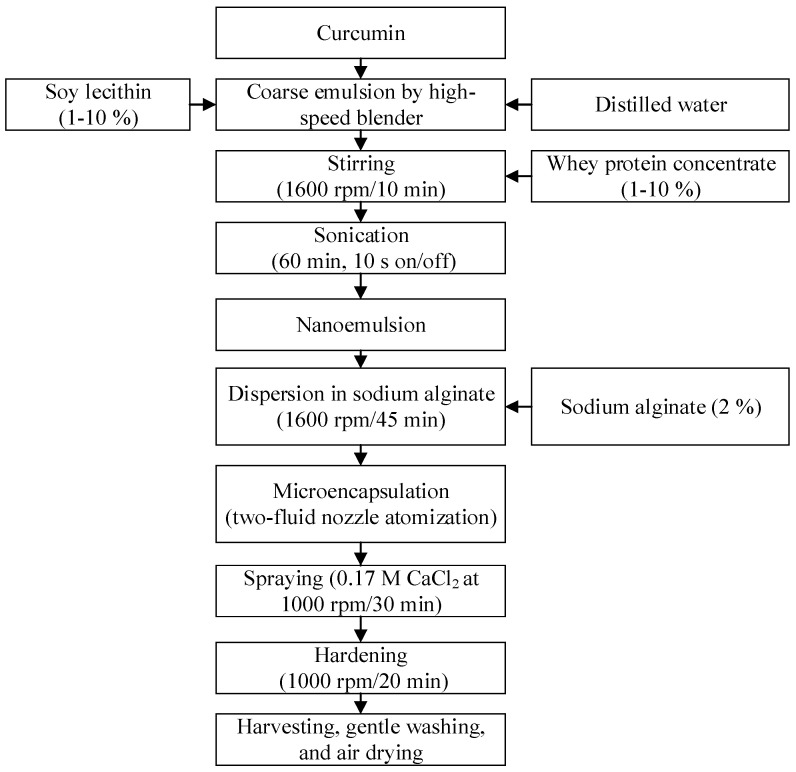
Preparation of CHBs by ionotropic gelation method. CHBs, curcumin hydrogel beads.

Liquid coarse emulsions were mixed with sodium alginate (2%) at 1600 rpm/45 min and sprayed by ionotropic gelation method with an in-house developed microencapsulator, which was patented by our research group [6,7]. The unit consisted of a peristaltic pump (flow rate of 80 mL/min), orifice with inner and outer nozzles (inner nozzle diameter: 1 mm with 1.30 mm annular space (carrying pressurized air) between inner and outer nozzle), air compressor (0.75 bar) and magnetic stirrer. The experimental process is illustrated in Figure 1 and the ionotropic gelation process was conducted at room temperature. The hardened samples were sieved, washed with deionized water to remove the residues of unreacted CaCl_2_, and then air dried. The samples were stored at room temperature in a laboratory vacuum-desiccator until further experimental analysis (usually within 2 weeks).

### 2.5. Characterization

#### 2.5.1. Morphology and Colour

Compound microscope (Motic, Kowloon, Hong Kong) with a calibrated stage micrometer at 4× magnification (Motic EF-N Plan 4× lens) was used to determine the size distribution of 10 random CHBs. BioVis Image Plus (Expert Vision Labs Pvt. Ltd., Mumbai, Maharashtra, India) software was used to determine the size distribution of the CHBs after calibration. The colour values (L, a, b, and ΔE, representing intensities of lightness, redness, yellowness, and colour difference, respectively) among the control as well as samples were measured using a colorimeter (MiniScan XE Plus, HunterLab, Reston, VA, USA) after calibration with a standard white plate. Three measurements were taken from each sample and the ΔE was calculated according to the Formula (1).

(1) $$\Delta E\;={\left[{\Delta L}^{2}+{\Delta a}^{2}+{\Delta b}^{2}\right]}^{\frac{1}{2}}$$

where, ΔL, Δa, and Δb = colour indices

#### 2.5.2. Encapsulation Efficiency (%)

Encapsulation efficiency was determined from the amount of curcumin actually encapsulated in CHBs (determined by UV–VIS spectrometry) with the initial amount of curcumin added to the emulsion. The encapsulation efficiency was calculated from the following Equation (2).

(2) $$\;\mathrm{Encapsulation}\;\mathrm{efficiency}\;\left(\%\right)=\left[\left(\frac{\mathrm{Actual}\;\mathrm{amount}\;\mathrm{of}\;\mathrm{curcumin}\;\mathrm{content}\;\mathrm{in}\;\mathrm{CHBs}\;\left(g\right)}{\mathrm{Initial}\;\mathrm{amount}\;\mathrm{of}\;\mathrm{curcumin}\;\left(g\right)}\;\right)\times 100\right]\;\;$$

Here, the actual loading content was measured by subtracting free curcumin content to the initial loading content according to Equation (3).

(3) Actual curcumin content in CHBs (g)=[Initial amount of curcumin (g)−free curcumin content (g)]

Free curcumin content was calculated by taking absorbance at 425 nm of the CaCl_2_ solution after harvesting using a double beam spectrophotometer (UV 1800, Shimadzu, Kyoto, Japan).

#### 2.5.3. Fourier Transform Infrared (FTIR) Spectroscopy

The interaction among the materials was determined at room temperature by ATR-FTIR (Bruker BioSpin GmbH, Ettlingen, Germany) within the wavenumber range of 4000 to 400 cm^−1^ with 24 scans at a resolution of 2 cm^−1^.

#### 2.5.4. Scanning Electron Microscopy (SEM)

Samples were analyzed using SEM according to Patra and Sleem [11] with minor modifications. Both curcumin and blank HBs were placed on the SEM stubs and then sputter coated with gold under vacuum during 30 s. The SEM images were then taken under the voltage of 15 kV.

#### 2.5.5. Swelling Index

The swelling properties of CHBs were determined using phosphate buffer saline (pH 7.40) at 37 °C according to Equation (4) based on the study investigated by El-Gibaly [12].

(4) $$\mathrm{Swelling}\;\mathrm{index}=\left[\frac{{\mathrm{Swollen}\;\mathrm{weight}\;\mathrm{of}\;\mathrm{CHB}}_{s}-{\mathrm{initial}\;\mathrm{weight}\;\mathrm{of}\;\mathrm{dried}\;\mathrm{CHB}}_{s}\;}{{\mathrm{Initial}\;\mathrm{weight}\;\mathrm{of}\;\mathrm{dried}\;\mathrm{CHB}}_{s}}\right]$$

#### 2.5.6. In Vitro Release Behavior in Phosphate Buffer at pH 7.40

In vitro release behavior of CHBs was investigated according to Bisht, et al. [13] with minor modifications. Briefly, samples (200 g) were placed in phosphate buffer (20 mL, pH 7.40 ± 0.10) in a 50 mL conical flask over an orbital shaker at 37 °C. Aliquots were taken at predetermined intervals of time (i.e., every 30 min) and centrifuged at 3000 rpm for 10 min and 25 °C. The respective aliquots were immediately replaced with the same amount of fresh phosphate buffer. The precipitate (released curcumin) was immediately re-dissolved in ethanol and the absorbance was measured at 425 nm using a double beam spectrophotometer. The concentration of curcumin was calculated using curcumin standard curve and the released curcumin (%) was calculated according to the Equation (5).

(5) $$\mathrm{Release}\;\left(\%\right)=\left[\left(\frac{{\left(\mathrm{Curcumin}\right)}_{\mathrm{rel}}}{\;{\left(\mathrm{Curcumin}\right)}_{\mathrm{tot}}}\;\right)\times 100\;\right]\;\;\;$$

where, [Curcumin]_rel_ = concentration of released curcumin at time t and [Curcumin]_tot_ = total amount of curcumin.

### 2.6. Preparation of CHBs-Fortified Functional Kulfi

**Figure 2:**
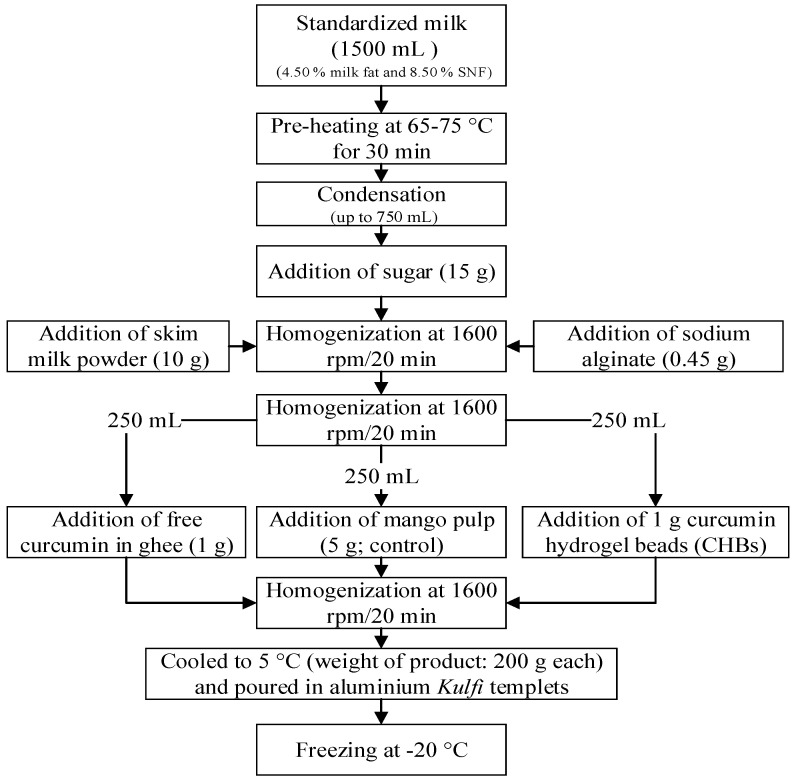
Development of functional *Kulfi* fortified with CHBs. CHBs, curcumin hydrogel beads; SNF, solids-not-fat.

The CHBs-fortified functional *Kulfi* was prepared according to Rohini, et al. [9] and the methodology is illustrated in Figure 2. Samples were coded as control (*Kulfi* fortified with mango pulp), curcumin *Kulfi* (*Kulfi* fortified with free curcumin (1 g) without mango pulp), and CHBs-fortified *Kulfi* (*Kulfi* fortified with microencapsulated curcumin (1 g)). All the experiments were performed in three independent determinations.

### 2.7. Physical Characteristics

#### 2.7.1. Colour

The colour values (L, a, b, and ΔE) among the control as well as samples fortified with free curcumin and CHBs were measured using a colorimeter and the ΔE was calculated according to Equation (1).

#### 2.7.2. Melting Rate

The melting rate of functional *Kulfi* was analyzed by the method according to Giri, et al. [14]. Briefly, a sample (50 g) was placed on a wire screen (6 holes/cm) over a funnel that was attached to a food-grade conical flask (500 mL). The time (min) taken by the sample for melt-down and dripped volume was recorded at 26 ± 1 °C. The melting rate was expressed as mL/min.

### 2.8. Microbiological Analysis

Generally, food safety management of any final food product is an important strategy, especially dairy-based food products like functional *Kulfi*. This dairy-based product being a nutritious food may serve as a good medium for the microbial growth that can cause the spoilage of the food product; thus, we performed microbiological analyses of functional *Kulfi* using pour plate method. Briefly, sample (11 g) was aseptically mixed with 99 mL sterile water and the mixture was homogenized, followed by serial dilutions (10^−1^ to 10^−6^) containing 0.90% sodium chloride. All the samples were determined for standard plate count (SPC), coliform, as well as yeast and mold counts according to an Indian Standards Institute method [15]. Microbiological analyses were recorded after incubation for 24 h (SPC and coliform) and 48 h (yeast and mold) and expressed as colony forming units (CFUs) per mL.

### 2.9. Sensory Evaluation

Sensory evaluation of functional *Kulfi* was performed with 15 trained voluntary panellists (7 female and 8 male), having prior experience in sensory profiling and were completely familiar with dairy sensory attributes. Panelists evaluated the colour & appearance, body & texture, flavor & taste, melting rate, and overall acceptability of the control and *Kulfi* fortified with free curcumin (1 g) and CHBs (1 g). Marketed *Kulfi* was compared with *Kulfi* fortified with CHBs.

### 2.10. Statistical Analysis

All experiments were performed at least in triplicate and the data was presented as an average ± standard deviation (SD). Data were subjected to statistical analysis by analysis of variance (ANOVA) and Duncan’s multiple range tests at *p* < 0.05 using GraphPad Prism^®^ 5.0 (GraphPad Software, San Diego, CA, USA). Graphs were constructed using Microsoft^®^ Office Professional Plus 2019 (Microsoft Co., Ltd., Redmond, WA, USA).

## 3. Results and Discussion

### 3.1. Particle Size and Apparent Viscosity

The hydrodynamic radius and polydispersity index of curcumin emulsion were found to be 2152.33 ± 374.16 nm and 0.349 ± 0.02, respectively (data not shown). The apparent viscosity of curcumin emulsion with alginate decreased from 0.90 to 0.37 Pa·s with increase in the shear rate (data not shown), indicated non-Newtonian shear thinning (pseudoplastic) behavior; however, the apparent viscosity of the curcumin emulsion without alginate and control (emulsion without curcumin and alginate) increased from −0.00089 to 0.000429 Pa·s and −0.0076 to 0.000162 Pa·s, respectively. A recent study demonstrated the increase in viscosity when polysaccharide was added to emulsion samples [16]. This proved the role of alginate in changing the viscosity of emulsion samples due to its high molecular weight or the formation of a gel network. This kind of behavior was the most common type of non-ideal behavior exhibited by emulsions.

### 3.2. Characterization of CHBs

#### 3.2.1. Size of the CHBs and Color

The size of 10 randomly selected fresh CHBs from different batches was found to be in the range of 1349.60 to 1834 μm, whereas after 24 h of drying at room temperature, the CHBs size was about 753.35 to 1120.25 μm. The average particle size of the fresh and dried CHBs was found to be 1393.58 ± 175.44 μm and 889.71 ± 191.19 μm, respectively. It seems possible that the encapsulation process might have influenced on the variation of CHBs particle size. A study documented the encapsulation method (i.e., air atomization technique), concentration of core & wall material, and air pressure had an influence on the size distribution of alginate poly-l-lysine microparticles [17]. Another study optimized the minimum value of air and liquid pressure to be 0.50 bar and 0.60 bar to ensure the flow break up, thereby formation of smooth and micronized capsules [18]. In the same study, it was reported that the air and liquid pressure were close to 1 at high and low pressures, microcapsules had a small size, while air and liquid pressure changed to intermediate values resulted a bigger microcapsules [18]. Likewise, Cui, et al. [17] observed the slightly decreased microcapsule particle size when sprayed at too short or too long a distance. Thus, atomization conditions may play a vital role in production of different size HBs.

The ΔE value was found to be 32.00 ± 1.88, which indicated a great difference in the colour, was observed between the curcumin and control CHBs (Table 1). The ‘a’ and ‘b’ values were significantly higher in CHBs (19.95 ± 0.30 and 46.46 ± 2.71) when compared to the control HBs (8.41 ± 0.30 and 28.74 ± 0.65). This striking difference between control and CHBs might be related to fluorescent yellow colour of curcumin, which contributed to intense increase in the a (red to green) and b (blue to yellow) parameters. The L value was higher in control HBs (62.24 ± 1.00), which represented a whiter colour as compared to CHBs (38.31 ± 0.29). The pale yellow to white colour in control HBs could be ascribed to presence of whey protein concentrate and alginate, while darker colour of CHBs due to the dominating presence of curcumin (Supplementary Figure S1). Moreover, slight yellow colour of control HBs may be due to the presence of lecithin [19] and probably whey protein fraction [20].

#### 3.2.2. Encapsulation Efficiency (%)

The encapsulation efficiency of CHBs was found to be 95.04 ± 2.08%. The high encapsulation of curcumin could be related to the combination of lecithin, whey protein concentrate, and calcium alginate, which collectively stabilized the curcumin within the CHBs. The high encapsulation efficiency could be related to curcumin-sodium alginate interactions, such as hydrophobic-hydrophilic or hydrophobic-hydrophobic interactions [21] due to aromatic rings of curcumin and hydrophobic regions of wall material. Moreover, H_2_ bonding between the carboxyl groups of the wall material and the hydroxyl groups of the curcumin may play a vital role in binding the curcumin and wall material [22]. There are, however, other possible explanations including interaction of the sodium and calcium ions that may form the less rigid network, thereby high encapsulation efficiency [23]. Additionally, the increase in encapsulation efficiency ascribed to the use of stabilizer, whey protein concentrate that stabilized the curcumin during the emulsion preparation process [24]. Similarly, a study reported the increased encapsulation efficiency of vitamin D_3_ loaded nano-niosomes with increase in different stabilizing agents [25]. These findings were in line with studies that reported high encapsulation efficiency (83 to 97%) for sodium caseinate [26], zein [27], and Persian gum based curcumin capsules [22]. Our findings suggested that the high curcumin encapsulation efficiency could increase the loading of active ingredients in the particles and further provide a stability against oxidation.

#### 3.2.3. Fourier Transform Infrared (FTIR) Spectroscopy

**Figure 3:**
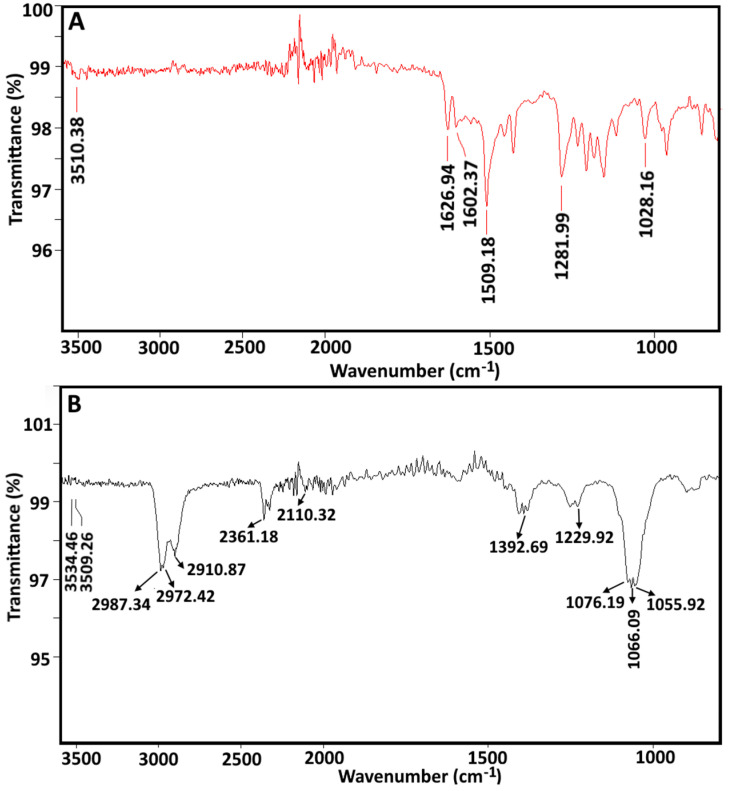
FTIR spectrum of samples: pure curcumin (**A**) and CHBs (**B**). FTIR, Fourier transform infrared; CHBs, curcumin hydrogel beads.

In the spectrum of pure curcumin (Figure 3A), the characteristic peak at 3510 cm^−1^, corresponds to the –OH stretching vibration of curcumin. Peak at 1627 cm^−1^ corresponds to stretching vibration of *v*(C=C) and *v*(C=O) characters, and at 1602 cm^−1^ attributed to symmetric aromatic ring stretching vibrations of *v*(C=C). Similar peaks were recorded at 1626 cm^−1^ for *v*(C=C) as well as *v*(C=O), and at 1601 cm^−1^ for *v*(C=C) by Kolev, et al. [28] and Bich, et al. [29]. The peak at 1509 cm^−1^ was assigned to C=O vibrations, peak at 1281 cm^−1^ attributed to the aromatic *v*(C–O), peak at 1028 cm^−1^ was assigned for *v*(C–O–C) and peak at 1428 cm^−1^ was attributed to the olefinic (C–H) bending vibrations, which are quite similar to the previously reported work [30,31].

The FTIR spectrum of CHBs (Figure 3B) observed with peak at 3509 cm^−1^ (pure curcumin spectra), corresponds to the −OH, which was narrower/disappeared after encapsulation. The presence of a narrowed band could be explained by the addition of alginate −OH and (C(=O) OH) groups, which formed the chelating structure and loss of H_2_ bonding between −OH functional groups. Similarly, These results further support the FTIR analysis investigated by Daemi and Barikani [32], where authors concluded the narrower of −OH peak was due to the presence of calcium alginate in CHBs. The stretching vibrations of the aliphatic C–H group shifted towards higher wave numbers (2987 to 2910 cm^−1^) in CHBs when compared to free alginate (2925 to 2854 cm^−1^).

For the sodium alginate samples, asymmetrical and symmetrical stretching (C(=O)OH) bonds were detected at 1609 and 1416 cm^−1^, which were documented in our previous investigation [8]. Moreover, stretching vibrations of aliphatic C–H groups were found at 2925 to 2854 cm^−1^ [32]. Moreover, polysaccharide structural groups, including C–O and C–O–C stretching were recorded at 3438 cm^−1^ [33], 1302 cm^−1^, 1095 cm^−1^, 1030 cm^−1^, and 947 cm^−1^.

The stretching vibrations of primary and secondary hydroxyl groups were also present at 1076 to 1055 cm^−1^ [34]. The peaks in CHBs demonstrated a disappearance of carboxylate ion at 1609 cm^−1^ in sodium alginate, while shifted carboxylate ion was observed from 1416 cm^−1^ to 1407–1381 cm^−1^, indicated the effect of sodium-calcium ions replacement in CHBs. Moreover, a possible explanation for the shift in wavenumber may be the charge density, radius, and atomic weight of the cations (Ca^2+^).

### 3.3. Scanning Electron Microscopy (SEM)

**Figure 4:**
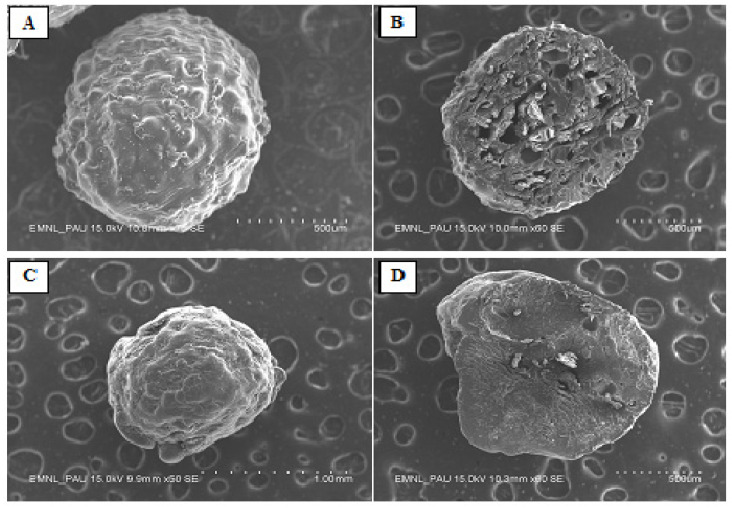
Microstructure of hydrogel beads (HBs): blank HBs (**A**) at 500 µm, cross-sectional view of blank HBs (**B**) at 600 µm, CHBs (**C**) at 100 mm, and cross-sectional view of CHBs (**D**) at 600 µm. CHBs, curcumin hydrogel beads.

The SEM microphotographs of the blank and CHBs are shown in Figure 4. The capsules formed the agglomerated structure with spherical shape, smooth, and covered with coating material. Similar studies were reported by Patra and Sleem [11] on the encapsulation of curcumin developed by poly (l-lysine) trisodium citrate and silica sol. Microcapsules were loaded with curcumin, which modified the surface appearance and became smooth [35]. There is no crack or fissure on the surface of CHBs. Similar reports were documented by Cano-Higuita, et al. [36], in which external surfaces demonstrated the existence of solid walls with no cracks or breaks. This indicated that the developed CHBs were suitable for improved protection and retention of curcumin. Blank samples were rough on the surface due to small inward dents that may have collapsed the HBs wall material [12]. Similar results were observed by Nayak, et al. [37] and concluded the presence of an uneven or irregular surface with a continuous wall and small pores on the outer wall of the HBs. It seems possible that these results were due to loss of water molecules that resulted in the shrinkage of the polymeric gel. 

### 3.4. Swelling Index

The average swelling index of CHBs was found to be 10.21 to 37.92 from 2 to 12 h in PBS at pH 7.40. It signifies the CHBs were 37.92 times swelled from its initial size after 12 h in PBS at pH 7.40. The drastic increase in the size of CHBs was observed from 0 to 6 h. Generally, the release behavior of encapsulated compounds is controlled by matrix swelling, hydration, and polymer dissolution. Moreover, in this study, the curcumin release is affected by the rate of water uptake (swelling rate) and the diffusion rate of the curcumin through the swollen gel. A study by Chen, et al. [38] reported that the swollen systems may increase gastric retention times, promote drug absorption within the stomach, and increase controlled release rate of active substances in the gastrointestinal tract. Another study by Arza, et al. [39] developed a swellable and floatable gastroretentive drug delivery system and concluded that the high ability to swell showed a better gastroretentive abilities and sustained drug release at a target site. Therefore, the swelling capacity in our study may promote the high release of CHBs in the gastrointestinal tract.

### 3.5. In Vitro Release Behavior in Phosphate Buffer at pH 7.40

**Figure 5:**
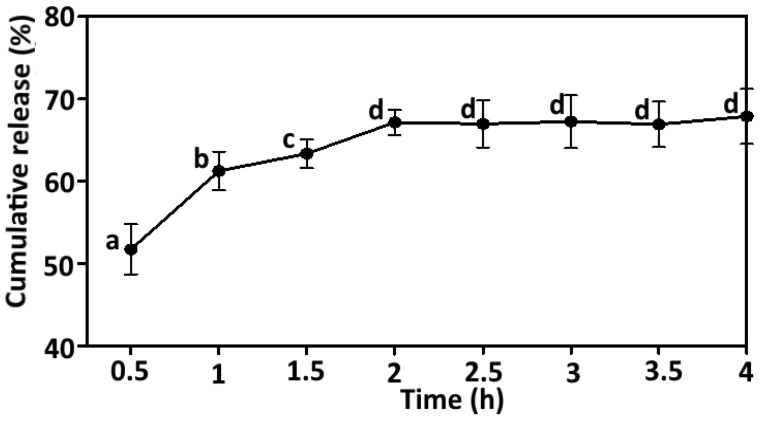
Cumulative release (%) of curcumin from CHBs in phosphate buffer at biological pH 7.40. Error bar represents the standard deviation of the mean determined from three independent determinations. Different lower-case alphabets (a to d) represent significant differences (*p <* 0.05; Duncan’s multiple range test) over the time (0.50 to 4 h). CHBs, curcumin hydrogel beads.

Curcumin was released at a faster rate of 51.72 ± 5.29% after 30 min and it was gradually increased up to 67.15 ± 2.65% after 2 h (Figure 5). However, Sari et al. (2015) concluded that over 90% of the nano-encapsulated curcumin using whey protein was retained in emulsion during simulated gastric digestion (2 h). This may be due to the presence of calcium alginate on the outer surface of CHBs in our work, which was easily solubilized and facilitated enhanced bioavailability. The % release of curcumin was maximum after 2 h and then the % cumulative release was increased slightly up to 67.88 ± 5.81 % after 4 h in the phosphate buffer at biological pH (7.40) as shown in Figure 5. This may be due to the solubility of the core material in the phosphate buffer (pH 7.40). In contrast, Tsai, et al. [40] reported the slow release of curcumin nanoparticles loaded in poly(lactic-co-glycolic acid) with biphasic releasing pattern and 59% release occurred after 12 h, which further increased up to 89% at the end of 6 days. The findings further in lined with the earlier study, in which microencapsulated curcumin in crosslinked jelly fig pectin exhibited a cumulative release of 95.34% over 24 h [41]. Similarly, Reddy, et al. [23] observed the high release of curcumin (45 to 65%) encapsulated in mixture of sodium alginate/montmorillonite into the dissolution medium at pH 7.40. A report by Govindaraju, et al. [21] prepared a 0.20% polysorbate 80 and alginate based nanosuspensions and reported a high cumulative release in simulated colonic fluid within 24 h due to high digestibility of alginate under colonic microflora. A more recent study documented the curcumin release of 43% at 120 min in the short gastric residence time and 16% at 180 min in the long gastric residence time when curcumin-whey protein microparticles enriched in yogurt [42].

Generally, curcumin becomes a hydrophilic with high water solubility under alkaline conditions due to deprotonation of hydroxyl groups [43]. In phosphate buffer at pH 7.40, the cross-linking network may rupture and facilitate the water permeation into the sodium alginate, thereby swelling of CHBs and then diffusion of curcumin into the dissolution medium [21]. Another possible explanation for the higher release rate in phosphate buffer could be related to the less interaction of carboxylic groups with phosphate buffer, allowing the network to be loose that can facilitate the leaching of curcumin from the network into the dissolution medium [23]. The use of lecithin as an emulsifier also contributed to the increase in solubility of curcumin in alkali conditions. These results implied that the calcium-alginate not only protected the curcumin but also controlled the release of curcumin under in vitro gastrointestinal conditions. Moreover, CHBs could release curcumin more than 60% within 6 h in human intestinal conditions. Thus, the in vitro release showed no immediate burst effect, indicating that the CHBs was mainly driven by a diffusion-controlled mechanism, which can be useful in the controlled release applications.

### 3.6. Physical Charecteristrics of Functional Kulfi

#### 3.6.1. Colour

The increase in the L value (73.03 to 75.88) showed insignificant differences (*p* > 0.05) in all samples, while there were significant differences (*p* < 0.05) in a (−1.40 to −4.97), b (22.43 to 59.30) and the ΔE (11.60 to 27.07) values for both samples fortified with curcumin compared to a control (a = −0.80 and b = 33.04) as shown in Table 2. Generally, surface colour depends on the composition and other characteristics, such as ingredients and processing conditions. The higher L value for all samples might be related to the presence of ice crystals that reflected the light before melting the samples. The samples with curcumin showed a higher average a value (a = −4.97) compared to control (a = −0.80) and the sample fortified with CHBs (a = −1.40). Likewise, a sample with curcumin had a significantly (*p* < 0.05) higher b value (559.30) than that of control (b = 33.04) and the sample fortified with CHBs (b = 22.43). The higher a and b values for samples with curcumin are likely to be related to the free availability of curcumin. The sample with curcumin had a higher average ΔE value (ΔE = 27.07) compared to the sample fortified with CHBs (ΔE = 11.60). Obviously, this was expected due to the presence of curcumin, which was in yellow colour. Similarly, it was observed that the encapsulation played a vital role in holding the curcumin samples that effectively protected the encapsulated curcumin extract from release. In accordance with the present results, a previous study by Park, et al. [44] demonstrated the change in colour values by the addition of turmeric in dairy foods. Similarly, another study showed the homogeneity in colour values for nanoencapsulated curcumin fortified in yogurt [45]. Herein, encapsulation masked the colour of samples (pale metanil yellow) compared to unencapsulated control (dark yellow), indicating the use of encapsulation in development of curcumin enriched dairy foods.

#### 3.6.2. Melting Rate

The melting rate of the functional *Kulfi* samples is shown in Table 3. Generally, the melting rate of dairy products is influenced by factors, such as physical structure, ice crystal size, fat network, air penetration, and ice phase volume [46]. All the samples insignificantly (*p* < 0.05) showed the low melting rate (<0.89 mL/min), which agreed with a previous study by Muse and Hartel [46], in which authors reported a lower melting rate (<1 mL/min) for three ice-cream formulations. In general, presence of total soluble solids has been shown to influence melting rate. For example, Salama [47] concluded that the presence of sugar (ranged from 20 to 60%) resulted in a reduction in the melting rate of ice cream. Generally, total soluble solids are related to the presence of mixed sugars (carbohydrates), which have high water-holding capacity, thereby contributing to a lower melting rate [10]. In frozen storage, the *Kulfi* samples may undergo partial coalescence, where clumps and clusters of the fat globules may form a strong network by trapping air within the fat and other components, hence decreased the melting rate of samples [48]. However, extremely high concentration of fat content may cause faster meltdown of samples. In our study, addition of curcumin (1 g) and CHBs (1 g) showed changes in melting rate of samples fortified with curcumin and CHBs. A study by Giri, et al. [14] highlighted the decreased melting rate as influenced by the addition of stevia (0.05 to 0.06%), while another study by Prindiville, et al. [49] stated the increased melting rate with increased fat content in chocolate ice cream. Overall, the study showed that curcumin fortified samples could melt slower than normal melting rate, thus promoting the good quality and body of dairy based frozen food formulations using microencapsulation.

### 3.7. Microbiological Analysis

The microbial growth (yeast and mold), including SPC and coliforms, of all samples is shown in Table 3. Samples fortified with curcumin and CHBs resulted in a significant reduction (*p* < 0.05) of yeast and mold growth. The SPC in functional *Kulfi* samples fortified with curcumin and CHBs, respectively recorded as 17.84 × 10^4^ CFU/mL and 13.77 × 10^4^ CFU/mL, which was >8 and 11-times lower than the control (154.70 × 10^4^ CFU/mL), respectively. Similar tendency was observed in coliforms for samples fortified with curcumin and CHBs. It was obvious to notice a lower microbial growth due to the well-known anti-microbial properties of curcumin against a wide range of food spoilage bacterial species [50]. A study highlighted the enhanced anti-microbial efficacy of curcumin after microencapsulation [51], which could be related to controlled-release of curcumin on the microbial cell surface and then penetration into cell membrane as well as tissues [52] by the quorum sensing (QS) system, in which curcumin exert an inhibitory effect on the bacterial biofilm formation process (an aggregation of microbial tissues wrapped in bacterial extracellular macromolecules) [53]. According to the quality criteria assigned by Indian Standards Institute [15], the acceptable bacterial growth for coliform and total bacteria count must be <100 CFU/g and 2.50 × 10^5^ CFU/g, respectively. Moreover, the high microbial growth in the control sample could be related to rapid melting and other favourable conditions that might have contaminated the samples. Therefore, our findings reported the lower microbial count than the prescribed limits and thus these samples could be considered safe for consumption. These findings broadly supports the investigation conducted by Wang, et al. [51], where authors highlighted the high anti-bacterial and anti-fungal efficacy of the encapsulated curcumin than unencapsulated curcumin. This could be related to microencapsulation, which enhanced the anti-bacterial and anti-fungal efficacy of the encapsulated curcumin by controlled release into the cell membrane of pathogens, ultimately causing cell death [10]. In brief, the findings concluded the lower viable counts of microorganisms in functional *Kulfi* supplemented with encapsulated curcumin, indicating the potential application of microencapsulation in development of curcumin enriched dairy products.

### 3.8. Sensory Evaluation

**Figure 6:**
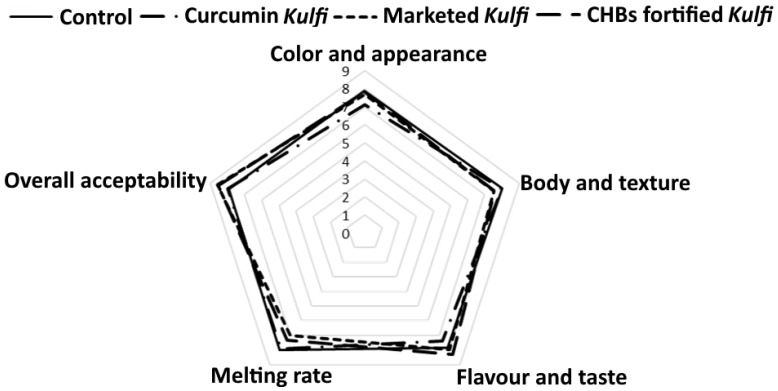
Sensory analysis of *Kulfi* fortified with CHBs. Hedonic scale: 9 = like extremely, 5 = neutral, and 1 = dislike extremely; CHBs, curcumin hydrogel beads.

Mean sensory scores for control, marketed *Kulfi*, and samples fortified with curcumin and CHBs are illustrated in Figure 6. Samples fortified with curcumin and CHBs showed significant differences (*p* < 0.05) in colour and appearance as well as flavour and taste along with overall acceptability when compared with the control sample (Figure 6). Samples fortified with curcumin scored lower values for colour and appearance (7.13) than that of control (7.86) and the sample fortified with CHBs (7.83). These results could be related to the decolorization of curcumin and colour shift during processing, which was in line with our visual observation and instrumental colour of samples. Interestingly, the body and texture of samples fortified with CHBs had a lower score (7.60) that of control (8) and samples fortified with curcumin (8), which may relate to the presence of CHBs that influenced the visual texture of the sample. Sample supplemented with curcumin resulted in slightly lower values for flavour and taste (7.36) that of control (7.83) and sample fortified with CHBs (8.33). The low flavour and taste values could be attributed to the presence of free curcumin, while high values might be related to the encapsulated curcumin in the sample.

Melting rate score showed a no significant difference (*p* < 0.05) among the samples (~8) as shown in Figure 6. Sample fortified with CHBs reported the high overall acceptability (8.56), followed by sample supplemented with curcumin (7.96) and control (7.93). Moreover, samples fortified with CHBs had similar sensory attributes as marketed *Kulfi,* demonstrating the high similarity in acceptance of *Kulfi* fortified with CHBs in the consumer market. Overall, fortification of CHBs showed high acceptable sensory properties, which agreed with a previous investigation by de Campo, et al. [54], in which authors observed that the addition of zeaxanthin nanoparticles in yogurt showed a no effect on the overall acceptability of the yogurt samples. Shehata and Soliman [55] prepared caseinate-curcumin nanoparticles and fortified in functional yogurt with improved body and textural properties, thereby received a high consumer acceptability. In another study, Ershadi, et al. [22] prepared Persian gum based curcumin nanoparticles and incorporated in Indian traditional dairy product, *Kefir* (1.50% of curcumin-loaded nanoparticles). This study reported a high consistency of *Kefir* due to addition of curcumin-loaded nanoparticles and showed the reduced low-density lipoprotein, total cholesterol, and triglycerides in the serum of rats fed with fortified *Kefir* samples. Kumar, et al. [26] formulated sodium caseinate based curcumin nanocapsules and fortified in development of functional ice-cream. The fortified functional ice-cream exhibited no significant difference in sensory attributes between the control and ice-cream fortified with curcumin nanocapsules. Likewise, Borrin, et al. [56] prepared the pineapple ice-cream enriched with curcumin-loaded nanoemulsions without significant change in sensory attributes of ice-cream fortified with or without curcumin-loaded nanoemulsions. Previous studies have stated that the micro and/or nanoencapsulation has a feasibility to be used in food fortification without affecting their sensory acceptability and quality attributes [8,44]. Moreover, panellists were further complemented for receiving no curcumin smell, indicating that the microencapsulation contributed to mask the curcumin smell in samples supplemented with CHBs.

## 4. Conclusions

Ionotropic gelation method for encapsulating curcumin in the form of hydrogel beads found a suitable delivery vehicle for encapsulation. The microphotographs indicated that the hydrogel beads were spherical, discrete, and completely covered with coating material. The good % release of curcumin indicating that the controlled release under simulated gastrointestinal conditions. Sample fortified with CHBs showed a negligible yellowness due to the presence of encapsulated CHBs. Microbiological analyses showed a high reduction for both the samples supplemented with curcumin and CHBs. Sensory analysis of samples supplemented with CHBs reported a high score for colour & appearance, flavour & taste, and overall acceptability, which was particularly related to the use of microencapsulation. Therefore, our results showed the possibility of calcium-alginate material for the entrapment of curcumin by gelation method and their fortification in functional *Kulfi*. Moreover, functional *Kulfi* fortified with CHBs seemed to be used to improve colour and reduce microbial load of frozen dairy desserts.

## Figures and Tables

**Table 1:** Colour parameters of CHBs ^1^.

Sample	Colour Parameters			
	L	a	b	ΔE
Control HBs	62.24 ± 1 ^b^	8.41 ± 0.30 ^a^	28.74 ± 0.65 ^a^	-
CHBs	38.31 ± 0.29 ^a^	19.95 ± 0.30 ^b^	46.46 ± 2.71 ^b^	32 ± 1.88

^1^ All the data are mean ± standard deviation (*n* = 3) for three independent batches. Different superscript letters in each column mean significant differences (*p* < 0.05) from each other. HBs, hydrogel beads; CHBs, curcumin hydrogel beads; ΔE, total colour difference.

**Table 2:** Colour parameters of *Kulfi* fortified with CHBs ^1^.

Samples	L	a	b	ΔE
Control	73.03 ± 3.13 ^a^	−0.80 ± 0.14 ^b^	33.04 ± 2.93 ^b^	-
Curcumin *Kulfi*	75.31 ± 1.66 ^a^	−4.97 ± 0.21 ^a^	59.30 ± 2.68 ^c^	27.07 ± 6 ^a^
CHBs fortified *Kulfi*	75.88 ± 4.01 ^a^	−1.40 ± 0.31 ^b^	22.43 ± 1.05 ^a^	11.60 ± 2.10 ^b^

^1^ Results were expressed as a mean ± standard deviation (*n* = 3). Superscripts with lowercase letters in the same column are significantly different (*p* < 0.05, Duncan’s multiple range test) from each other. CHBs, curcumin hydrogel beads.

**Table 3:** Melting rate and microbiological analysis of *Kulfi* fortified with CHBs ^1^.

Samples	Melting Rate (mL/min)	Microbiological Analysis		
		Yeast and Mold (×10^3^ CFU/mL)	SPC ^†^ (×10^4^ CFU/mL)	Coliforms (×10^2^ CFU/mL)
Control	0.88 ± 0.29 ^a^	105.40 ± 1.57 ^a^	154.70 ± 5.07 ^a^	10.16 ± 0.74 ^a^
Curcumin *Kulfi*	0.89 ± 0.07 ^a^	11 ± 3.58 ^b^	17.84 ± 3.18 ^b^	0.74 ± 0.88 ^b^
CHBs fortified *Kulfi*	0.63 ± 0.23 ^b^	8.07 ± 2.94 ^c^	13.77 ± 4.55 ^c^	0.45 ± 0.91 ^c^

^1^ Results were expressed as a mean ± standard deviation (*n* ≥ 3). Superscripts with lowercase letters in the same column are significantly different (*p* < 0.05, Duncan’s multiple range test) from each other. CHBs, curcumin hydrogel beads; CFU, colony forming unit. ^†^ SPC = standard plate count.

## Data Availability

The data that support the findings of this study are available within the manuscript.

## Supplementary Materials

The following are available online at https://www.mdpi.com/article/10.3390/foods11020182/s1, Figure S1: Visual observation of hydrogel beads. Control HBs (A) and CHBs (B). HBs, hydrogel beads; CHBs, curcumin hydrogel beads.
