# Supplementary material for: Preparation of Curcumin Hydrogel Beads for the Development of Functional Kulfi: A Tailoring Delivery System
Source: Foods. 2022 Jan 11;11(2):182. doi: 10.3390/foods11020182 (PMC8774899; doi:10.3390/foods11020182)
Supplement: Supplementary file 1 [file foods-11-00182-s001.zip › foods-1536509-supplementary.pdf]

Supplementary Material

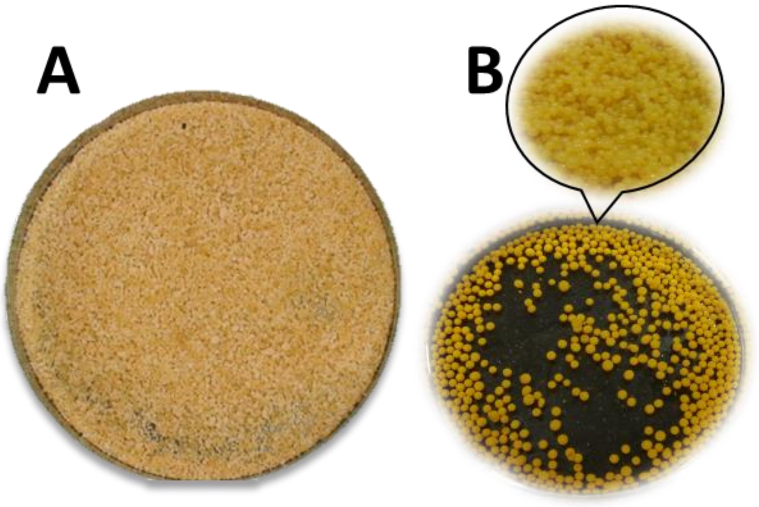

Figure S1. Visual observation of hydrogel beads. Control HBs (A) and CHBs (B). HBs, hydrogel beads; CHBs, curcumin hydrogel beads.
